# RIME-Net: A Physics-Guided Unpaired Learning Framework for Automotive Radar Interference Mitigation and Weak Target Enhancement

**DOI:** 10.3390/s26041277

**Published:** 2026-02-15

**Authors:** Jiajia Shi, Haojie Zhou, Liu Chu, Fengling Tan, Guocheng Sun, Yu Tao

**Affiliations:** 1 School of Transportation and Civil Engineering, Nantong University, Nantong 226007, China; shijj@ntu.edu.cn (J.S.); 2330310014@stmail.ntu.edu.cn (H.Z.); 2 College of Electronic and Information Engineering, Tongji University, Shanghai 201804, China; 3 School of Physical Science and Technology, ShanghaiTech University, Shanghai 201210, China; 4 Research & Design Institute, Sinohydro Engineering Bureau 8 Co., Ltd., Changsha 410004, China; 120204@powerchina.cn (F.T.); 119007@powerchina.cn (G.S.); 5 School of Electronic and Information Engineering, Suzhou University of Technology, Changshu 215500, China; taoyu@cslg.edu.cn

**Keywords:** FMCW millimeter-wave radar, range–Doppler map restoration, interference mitigation, weak-target enhancement

## Abstract

With the widespread deployment of automotive millimeter-wave radars, mutual interference and broadband noise severely degrade the signal-to-noise ratio (SNR) of range–Doppler (RD) maps, leading to the loss of weak targets. Existing deep learning methods rely on difficult-to-obtain paired training samples and often cause excessive target smoothing due to a lack of physical constraints. To address these challenges, this paper proposes RIME-Net, a physics-guided unpaired learning framework designed to jointly achieve radar interference mitigation and weak target enhancement. First, based on a cycle-consistent adversarial architecture, we designed the Interference Mitigation Network (IM-Net). IM-Net integrates spectral consistency loss and identity mapping constraints, learning a robust mapping from the interference domain to the clean domain without paired supervision, effectively suppressing low-rank interference and preserving signal integrity. Second, to recover target details attenuated during denoising, we propose the saliency-aware Target Enhancement Network (TE-Net). TE-Net combines multi-scale residual blocks and channel-spatial attention mechanisms, selectively enhancing weak target features based on saliency priors. Extensive experiments on diverse datasets show that RIME-Net significantly outperforms existing supervised and model-driven methods in terms of SINR, recall, and structural similarity, providing a robust solution for reliable radar perception in complex electromagnetic environments.

## 1. Introduction

The rapid advancement of autonomous driving and Advanced Driver Assistance Systems (ADAS) has imposed stringent requirements on the accuracy and reliability of vehicle environment perception, particularly for achieving continuous, blind-spot-free target detection under complex urban and adverse weather conditions [1]. Millimeter-wave Frequency Modulated Continuous Wave (FMCW) radar generates range–Doppler (RD) spectra by transmitting linear frequency-modulated signals and processing target echoes via Fast Fourier Transform (FFT), enabling simultaneous characterization of target range, velocity, and micro-Doppler features [2], whose quality directly determines the performance ceiling of perception systems [3]. Compared with optical sensors such as cameras and LiDAR, millimeter-wave radar is robust to illumination variations, rain, fog, and dust, while offering strong penetration capability and low cost, making it an indispensable sensing modality for intelligent driving systems [4,5]. However, with the large-scale deployment of vehicle-mounted millimeter-wave radars in intelligent transportation systems—especially in vehicle-to-vehicle (V2X) and cooperative driving scenarios—mutual radar interference has rapidly emerged as a critical bottleneck [6]. Synchronous transmission of frequency-modulated signals in adjacent frequency bands or overlapping time slots causes receiving radars to capture incoherent interference from neighboring vehicles, leading to spectrum competition and energy leakage [7]. Such interference manifests as ghost targets, full-spectrum energy fringes, and speckle-like noise in RD maps, severely degrading target distinguishability, reducing the signal-to-interference-plus-noise ratio (SINR), and increasing false alarm rates [8,9]. Persistent interference further introduces cumulative errors in velocity estimation and target tracking, potentially resulting in incorrect environmental perception and even catastrophic failures such as collisions in autonomous driving systems [10]. Consequently, effective suppression of radar mutual interference and reliable recovery of weak target signals have become urgent and fundamental challenges in automotive radar research.

In recent years, extensive research has been conducted on the mechanism analysis and modeling of millimeter-wave radar mutual interference to uncover its generation, propagation, and coupling principles [11]. It is widely recognized that signal overlap among different radars in the time, frequency, and spatial domains constitutes the fundamental cause of interference, with its severity jointly influenced by waveform design, modulation slope, antenna beam direction, and surrounding reflective environments [12]. Zhao et al. demonstrated through an interference propagation model that even microsecond-level timing offsets can cause significant energy diffusion in the RD domain due to nonlinear frequency and synchronization errors [13]. Kim et al. quantitatively analyzed interference energy diffusion and peak drift using time-frequency theory, revealing dynamic coupling characteristics between interference and target echoes in RD maps [14]. Wang et al. employed Monte Carlo simulations in multi-radar scenarios and identified modulation period differences and pulse overlap rates as key factors governing interference intensity and spatial distribution, further proposing a semi-empirical probability density model [15]. Li et al. showed that multipath scattering from buildings and large vehicles significantly amplifies interference power, resulting in an additional 6–10 dB signal-to-noise ratio degradation [16]. These studies establish a solid theoretical foundation and simulation framework for the development of interference suppression methods.

After the interference signal enters the receiver, it will undergo complex aliasing and phase disturbance with the real target echo in the frequency and time domains. After RD transformation, it will appear as artifacts of various shapes in the spectrum [17]. Specifically, strong interference often produces paired false spectral peaks near real targets, whose positions drift periodically with relative velocity and time offset, while moderate intensity interference forms energy fringes that penetrate the distance or Doppler dimension, masking nearby weak targets. However, dense multi-radar interference manifests as non-uniform elevation of the background noise floor, forming patchy noise patches [18]. The result is that interference may not only introduce false targets in the RD image, mislead subsequent detection algorithms based on constant false alarm rate (CFAR), causing a sharp increase in false alarm rate, but also mask the energy distribution of real weak targets, making it difficult to correctly identify and stably track small pedestrians, long-distance obstacles, or low radar cross-section (RCS) targets [19,20]. This dual negative effect can directly lead to a decrease in the reliability of perception systems in actual traffic scenarios, limiting the deployment and promotion of high-level autonomous driving functions [21].

Traditional signal processing and statistical modeling methods exploit the physical propagation mechanisms and statistical properties of radar signals to explicitly separate interference from target echoes using filtering, transform-domain analysis, and matrix decomposition [22]. By leveraging signal sparsity and separability in time-frequency or spatial domains, representative approaches include wavelet packet decomposition for multi-scale interference suppression [23], empirical and variational mode decomposition (EMD/VMD) for adaptive time-frequency separation in complex environments [24,25], and robust principal component analysis (RPCA) for low-rank background and sparse interference decomposition in RD images [26]. Recent enhancements combining VMD-based reconstruction and adaptive short-time Fourier filtering have achieved notable SINR gains in dense traffic scenarios [27,28]. Owing to their strong interpretability, implementation simplicity, and low computational complexity, these methods are well suited for resource-constrained in-vehicle platforms.

However, traditional signal processing methods exhibit inherent limitations in practical applications. These approaches usually rely on assumptions of signal stationarity and sparsity, which are often violated in high-speed dynamic scenarios due to target Doppler variation and time-varying interference characteristics [29]. In complex nonlinear and multi-source interference environments, their limited adaptability makes it difficult to preserve RD-map structural integrity and energy consistency, leading to weak-target masking and spectral distortion [30]. Moreover, decomposition- and filtering-based methods require careful parameter tuning, such as wavelet basis selection, EMD stopping criteria, and VMD mode number configuration, which are highly sensitive to interference conditions and lack self-adaptive capability [31]. Their ability to reconstruct complex RD patterns is also limited, causing severe performance degradation when interference power approaches or exceeds target power [32].

To overcome these limitations, deep learning methods adopt end-to-end data-driven modeling to learn complex nonlinear mappings between interference and targets for RD-map denoising and reconstruction [33]. Convolutional neural network–based approaches enable effective interference suppression by capturing local spectral correlations, significantly improving weak-target detection performance [34]. Multi-scale encoder–decoder architectures further enhance feature preservation by fusing high-resolution structural information during reconstruction [35]. Residual-learning strategies mitigate energy loss and detail blurring by modeling noise components rather than directly fitting clean signals [36]. Adversarial learning frameworks improve structural fidelity and contrast by enforcing distribution-level consistency between reconstructed and clean RD maps [37]. Recent studies have further enhanced robustness under low-SNR and complex interference conditions by incorporating prior guidance and long-range dependency modeling [38]. Expanded receptive-field designs based on dilated convolution and contrastive learning have also demonstrated notable improvements in structural similarity [39].

Despite their promising performance, purely deep learning–based methods face substantial challenges in real-world deployment. Most supervised approaches depend on large-scale paired clean and interfered RD datasets, which are extremely difficult to obtain in real traffic scenarios under identical spatiotemporal conditions [40]. The absence of explicit physical constraints and saliency guidance can lead to target blurring or energy over-smoothing, particularly when interference distributions deviate from the training domain [41]. In addition, the black-box nature of deep models limits interpretability, posing challenges for meeting stringent functional safety certification requirements in automotive systems [42]. Furthermore, the high computational cost of deep architectures makes it difficult to balance real-time performance and accuracy on resource-constrained in-vehicle platforms, even with model compression techniques [43].

Despite the effectiveness of existing learning-based radar interference mitigation methods, most of them rely heavily on paired clean-and-interfered training data. However, in real-world automotive radar scenarios, acquiring such paired data is extremely challenging, if not infeasible. Once interference occurs during radar signal acquisition, the corresponding clean reference signal cannot be simultaneously observed or retrospectively recovered under identical environmental and traffic conditions.

Although simulation-based approaches can synthetically generate paired data, they inevitably suffer from domain gaps, as simulated interference patterns and target responses cannot fully capture the diversity, randomness, and non-stationary characteristics of real-world multi-source radar interference. As a result, models trained on paired or simulated datasets often exhibit limited generalization performance when deployed in practical driving environments.

These practical constraints fundamentally limit the applicability of paired learning paradigms for automotive radar perception. Therefore, developing an unpaired learning framework that can effectively leverage unaligned clean and interfered data while incorporating physical prior knowledge becomes not only desirable but necessary for real-world automotive radar interference mitigation.

The main contributions of this paper are summarized as follows:

1. We propose a CycleGAN-based unpaired cross-domain mapping architecture with dual generators and dual discriminators, introducing cycle consistency, identity mapping, and spectral regularization to learn the transformation between interfered and clean RD domains.
2. We propose a physics-guided saliency reconstruction mechanism by incorporating propagation-model-based physical consistency constraints and saliency guidance to improve weak-target energy recovery and suppress background noise and spurious peaks during enhancement.
3. We develop a two-stage cooperative framework that integrates global domain mapping with local saliency enhancement, introducing cross-stage feature transfer and residual-consistency constraints to achieve hierarchical collaboration and feature fusion.

The remainder of this paper is organized as follows. Section 2 introduces the signal model of automotive FMCW radar and analyzes interference mechanisms, highlighting the characteristics of targets and interference in RD maps. Section 3 describes RD-map denoising and enhancement methods, including structure-consistent CFAR detection and target-preserving sparse wavelet denoising. Section 4 presents the proposed two-stage deep framework, including the CycleGAN-based interference suppression network and the saliency-guided TE-Net. Section 5 provides experimental results under mild interference, severe interference, and multi-target scenarios. Section 6 concludes the paper and discusses future directions. The code implementation is publicly available at https://github.com/programmerZhj/radar-denoise-enhance.git (accessed on 3 February 2026).

## 2. Automotive Radar Interference Modeling

### 2.1. FMCW Radar Signal Model

A fast linear FMCW radar transmitting Q consecutive chirps can be expressed as

(1) $$T_{s}(t)=\sum_{q=0}^{Q-1}s(t-qT)$$

where T is the pulse repetition interval and s(⋅) denotes the transmitted chirp signal.

The single chirp with normalized amplitude is

(2) $$s(t)=\exp(j2\pi (f_{c}t+0.5kt^{2}))\mathrm{rect}\left(\frac{t}{T}\right)$$

where $f_{c}$ is the carrier frequency and k=B/T is the chirp rate with sweep bandwidth B.

Here, we assume that the pulse repetition time equals the chirp duration, and rect(⋅) denotes the unit pulse that equals 1 on [0,1) and 0 otherwise.

For the q-th received chirp from a target, the time delay is τ, and the received signal is

(3) $$r_{q}(t)=A_{q}s\left(t-qT-\tau \right)+v_{q}(t)$$

where $A_{q}$ is the received amplitude, τ=2(D+νt)/c, and $v_{q}(t)$ is complex white noise.

Here, D and ν denote the target distance and radial velocity, respectively, and c is the speed of light.

After stretch processing, i.e., mixing $r_{q}(t)$ with the conjugated transmitted signal, the beat signal becomes

(4) $${\hat{y}}_{q}(t)=r_{q}^{*}(t)s(t-qT)$$

After low-pass filtering (LPF) and sampling with period $T_{s}$, each chirp produces P samples, and a target’s discrete beat frequency signal can be approximated as

(5) $$\begin{array}{c} {\hat{y}}_{p,q}\approx A_{q}\exp\left(j2\pi \kappa \frac{2D}{c}pT_{s}\right)\cdot \exp\left(j2\pi f_{c}\frac{2\upsilon }{c}qT\right)\\ \cdot \exp\left(j2\pi f_{c}\frac{2D}{c}\right)+v_{p,q}\;\;\;\;\;\;p\in \{0,P-1\}. \end{array}$$

Let ${\tilde{y}}_{p,q}$ denote additive in-band interference. For the discrete beat signal $y=y_{\left(qP+p\right)}$ with 0≤q≤Q−1, we have

(6) $$y_{\left(q\cdot P+p\right)}=\{\begin{array}{c} {\hat{y}}_{p,q}+v_{p,q}\;\;\;\;\;\;\;\;\;\;\;\;\;\;\;\;\;\;\;\;\;\;\;q\cdot P+p\in K\\ {\hat{y}}_{p,q}+{\overset{`}{y}}_{p,q}+v_{p,q}\;\;\;\;\;\;\;\;\;\;q\cdot P+p\notin K \end{array}$$

where K is the index set of interference-free samples.

The continuous beat signal is sampled at a period $T_{s}$, yielding P samples per chirp. By stacking Q consecutive chirps, we construct the discrete beat matrix $Y=\{y_{p,q}\}\in C^{P\times Q}$, where the indices p∈{0,…,P−1} and q∈{0,…,Q−1} represent the fast-time (range) and slow-time (Doppler) dimensions, respectively.

The range–Doppler map X is obtained by applying a two-dimensional Discrete Fourier Transform (2D-DFT) to the matrix Y:

(7) $$X=C_{P}\cdot Y\cdot C_{Q}^{T}$$

where $C_{P}\in C^{P\times P}$ and $C_{Q}\in C^{Q\times Q}$ are the DFT matrices of sizes P and Q, respectively. The first 1D-FFT across the fast-time dimension transforms the beat frequencies into range bins, while the second 1D-FFT across the slow-time dimension resolves the Doppler frequencies into velocity bins. The resulting magnitude spectrum |X| characterizes the spatial distribution and motion features of all detected targets.

### 2.2. Interference Mechanisms

The simplistic signal model described in Section 2.1 represents an idealized victim radar response. However, in dense V2X (Vehicle-to-Everything) scenarios, the range–Doppler (RD) map is often corrupted by asynchronous mutual interference from multiple adjacent radars. These interference signals typically exhibit varying modulation slopes and non-coherent time-frequency signatures, manifesting as “ghost targets” or full-spectrum energy fringes that penetrate the distance and Doppler dimensions.

Traditional signal processing methods, such as Wavelet packet decomposition or EMD, are built upon the assumption of signal-to-noise separability in specific bases. In complex, high-speed dynamic environments, these assumptions fail because interference energy often resides in the same frequency bins as weak targets, making linear filtering insufficient to suppress artifacts without attenuating true echoes, or the time-varying nature of radar mutual interference leads to non-stationary RD-map artifacts that fixed-parameter filters cannot adaptively track.

Millimeter-wave radar systems generate two-dimensional range–Doppler (RD) maps that characterize the spatial and motion properties of targets by transmitting frequency-modulated continuous waves (FMCW) and applying a two-dimensional Fast Fourier Transform (2D-FFT) to the received echoes. However, RD maps contain not only target reflections but also various types of interference-induced clutter and artifacts. Without effective suppression, these invalid or misleading components can easily result in false alarms, target misidentification, and even track loss.

Therefore, a thorough understanding of the fundamental structural differences between target signals and interference components in RD maps is essential for designing effective interference suppression and target enhancement algorithms.

In vehicular or multi-radar cooperative scenarios, interference may stem from radar-to-radar interference, multipath reflections, electromagnetic environmental interference, and system self-noise. Such interference typically manifests in RD maps as structured energy patterns such as “band-like,” “patch-like,” or “tiled” regions, which distinctly contrast with the sparse and localized nature of true targets. Their common characteristics—strong regularity, structural stability, concentrated energy, and high cross-channel correlation—allow them to be modeled mathematically as low-rank structures. Let an RD map frame be represented as a matrix $X\in R^{M\times N}$, where M and N denote the Doppler and range dimensions. Then, the interference component $X_{\mathrm{intf}}$ can be approximated as a low-rank matrix:

(8) $$X_{intf}\approx L,withrank(L)\ll min(M,N)$$

This indicates that interference can be reconstructed using only a small number of principal components (e.g., directional strip-like energy patterns), demonstrating strong redundancy. In practice, such interference appears as periodically recurring signals at fixed range or Doppler locations, horizontal or vertical strip-like strong echoes spanning the entire image, or artifact structures such as mirror images or false targets resulting from strong reflections. These structural properties support low-rank modeling and data-driven compression, which are commonly used in Robust Principal Component Analysis (RPCA) and deep low-rank networks. In contrast, true target echoes exhibit sparsity, locality, stability, and diversity in RD maps.

Thus, target signals can be mathematically modeled as a sparse matrix S, satisfying

(9) $$∥S{∥}_{0}\;\ll MN$$

where $∥\cdot {∥}_{0}$ denotes the number of nonzero elements. This sparsity indicates that only a small number of high-intensity pixels are required for detection and enhancement, while background pixels can be ignored.

It is worth noting that weak targets (e.g., distant pedestrians or motorcycles) have low reflection energy and are easily masked by interference, especially under large-area strong interference conditions. Therefore, accurately extracting sparse target structures while suppressing low-rank interference is a core requirement for deep network learning.

### 2.3. Sparse–Low-Rank Joint Modeling and Optimization

To address these fundamental limitations, we reformulate the restoration task as a joint sparse–low-rank optimization problem. By modeling interference as a globally distributed low-rank subspace and targets as localized sparse manifolds, we move beyond the simplistic pulse-echo model. RIME-Net acts as an approximate deep RPCA solver, where the neural architecture learns to distinguish complex non-linear interference signatures that lack an explicit closed-form mathematical expression. This deep learning approach provides the requisite non-linear capacity to restore high-fidelity target signatures in electromagnetic environments where standard techniques incur unacceptable performance degradation.

It is worth clarifying that the sparsity assumption adopted in this work does not imply that the number of targets is strictly limited. Instead, it refers to the fact that target responses in the Range–Doppler domain exhibit localized and structured energy concentration, in contrast to the globally distributed and broadband characteristics of interference.

Even in multi-target scenarios, target reflections tend to occupy limited and physically meaningful regions in the RD domain due to constraints imposed by range resolution, Doppler resolution, and radar waveform parameters. This form of local or structured sparsity provides a discriminative prior that can be exploited for interference mitigation and target enhancement.

Based on the above analysis, the observed range–Doppler (RD) image X can be modeled as the following structural decomposition:

(10) X=L+S+N

where L denotes the low-rank interference subspace, S represents the sparse target response, and N refers to Gaussian noise or background disturbance.

To extract S, a sparse–low-rank optimization framework (RPCA) can be adopted:

(11) $$\underset{L,S}{\min}∥L{∥}_{*}+\lambda ∥S{∥}_{1}\;s.t.X=L+S$$

where $∥\cdot {∥}_{*}$ denotes the nuclear norm that promotes low-rank matrix shrinkage, $∥\cdot {∥}_{1}$ is the $L_{1}$ norm that encourages sparsity, and λ is the trade-off parameter balancing the two regularization terms.

The trade-off parameter *λ* controls the relative contribution between the sparse target component and the low-rank interference component in the joint modeling formulation. Specifically, a larger *λ* enforces stronger sparsity on target responses, while a smaller *λ* places more emphasis on low-rank interference suppression.

In practice, *λ* is selected based on empirical observations to achieve a balanced separation between targets and interference. We find that *λ* values within the range of [0.1, 1.0] provide stable performance across different interference levels and target configurations. When *λ* is set too small, residual interference may remain, whereas excessively large *λ* values may lead to partial suppression of weak targets.

**Figure 1:**
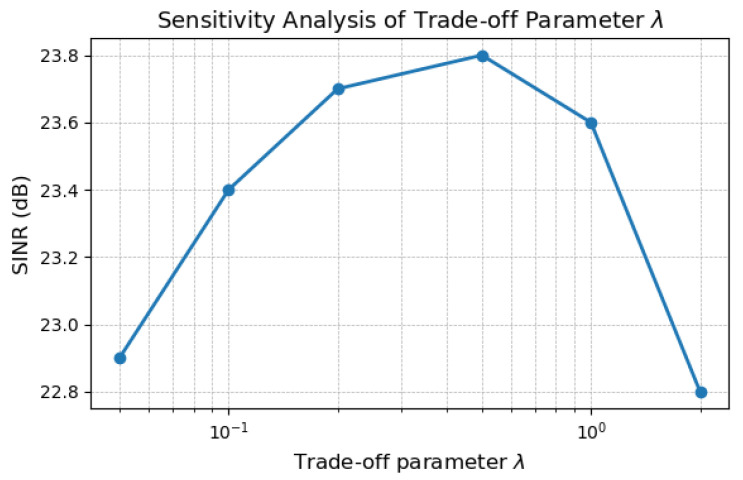
Sensitivity analysis of the trade-off parameter *λ*.

To evaluate the sensitivity of the proposed method to *λ*, we conducted experiments by varying *λ* within this range in Figure 1. The results indicate that the performance of the proposed framework remains relatively stable, with only minor fluctuations in quantitative metrics, demonstrating that the method is not overly sensitive to precise parameter tuning.

**Figure 2:**
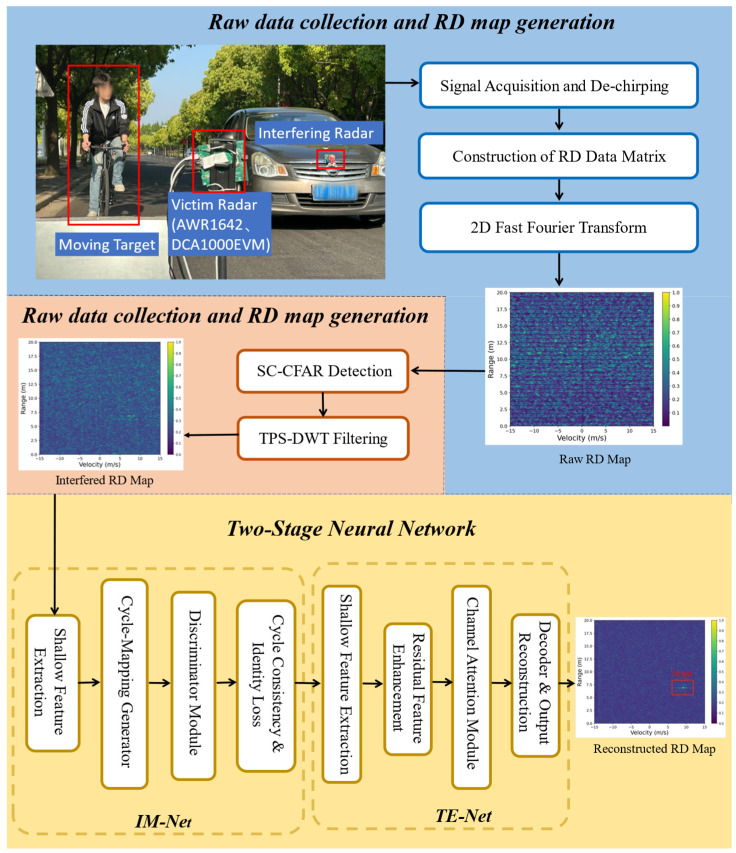
Overall System Pipeline.

The overall process of this article is shown in Figure 2.

## 3. Preprocessing Methods

### 3.1. SC-CFAR

Traditional constant false alarm rate (CFAR) detection methods estimate background statistics using a sliding window and apply a unified detection threshold, which implicitly assumes local background homogeneity. As a result, their detection performance degrades significantly in scenarios with strong background fluctuations, nonstationary clutter, or weak target responses, often leading to missed detections and unstable thresholding behavior. To alleviate these limitations, this paper introduces a structural-consistency enhancement term into the conventional CFAR framework and proposes an improved detection algorithm termed SC-CFAR. By explicitly modeling the local continuity and morphological aggregation characteristics of targets in the range–Doppler domain, the proposed method enhances the discrimination between true targets and interference-induced artifacts, enabling more robust detection under complex background conditions. Based on this enhanced detection mechanism, the overall algorithm framework is subsequently constructed, in which structural-consistency-aware detection serves as a critical component for guiding target localization and providing reliable priors for subsequent processing stages. The following subsection presents the overall processing flow of the proposed algorithm in detail.

**Algorithm 1:** Structure-Consistent CFAR (SC-CFAR)**Input:** RD Map $X\in R^{M\times N}$; background window B size $N_{w}$; local            neighborhood Ω (e.g., 5 × 5); intensity tolerance δ; base false            alarm rate $P_{fa}$; parameters α>0,γ∈[0,1]. **Output:** Binary detection map $D\in {\left\{0,1\right\}}^{M\times N}$ (1 = target, 0 = background).
 1:Initialize $D\leftarrow 0_{M\times N};$ 2:**for** each pixel (x,y) in X (excluding margin required by B and Ω) **do** 3:$\;\;\;\;\;\;\;B_{xy}\leftarrow$ set of background cells for sliding window around (x,y); 4:

$\;\;\;\;\;\;\;\mu_{n}\leftarrow \frac{1}{N_{w}}\sum_{(i,j)\in B_{xy}}X(i,j);$

 5:

$\;\;\;\;\;\;\;\sigma_{n}\leftarrow \sqrt{\frac{1}{N_{w}}\sum_{(i,j)\in B_{xy}}{\left(X(i,j)-\mu_{n}\right)}^{2}};$

 6:$\;\;\;\;\;\;\;\Omega_{xy}\leftarrow$ set of coordinates in local neighborhood centered at (x,y) 7:

       c←0;

 8:       **for** each $(i,j)\in \Omega_{xy}$ **do**  9:             **if** |X(i,j)−X(x,y)|<δ **then**  10:

             c←c+1

 11:          **end** 12:   **end** 13:

$\;\;\;C(x,y)\leftarrow c/\left|\Omega_{xy}\right|;$

 14:

$\;\;\;T(x,y)\leftarrow \mu_{n}+\alpha \cdot \sigma_{n}\cdot \sqrt{\frac{\ln\left(1/P_{fa}\right)}{N_{w}}}\cdot (1-\gamma \cdot C(x,y));$

 15:   **if** X(x,y)>T(x,y) **then**  16:

        D(x,y)←1;

 17:   **else** 18:

        D(x,y)←0

 19:   **end** 20:return D;


Given an RD map $X\in R^{M\times N}$, for each candidate pixel (x,y), the local background statistics within a sliding window are defined as

(12) $$\mu_{n}=\frac{1}{N_{w}}\sum_{(i,j)\in B}X(i,j),\sigma_{n}=\sqrt{\frac{1}{N_{w}}\sum_{(i,j)\in B}{\left(X(i,j)-\mu_{n}\right)}^{2}}$$

where B denotes the set of background cells and $N_{w}=|B|$.

A structural consistency measurement term is further introduced:

(13) $$C\left(x,y\right)=\frac{1}{\left|\Omega \right|}\sum_{\left(i,j\right)\in \Omega }1\left[\left|X\left(i,j\right)-X\left(x,y\right)\right|<\delta \right]$$

where Ω is a 5×5 neighborhood window, 1[⋅] is an indicator function for conditional evaluation, and δ is the intensity similarity threshold. This term measures the consistency between local pixels and the central pixel in terms of intensity and spatial continuity, reflecting the aggregation characteristics of the target region.

The final detection threshold is defined as

(14) $$T(x,y)=\mu_{n}+\alpha \cdot \sigma_{n}\cdot \sqrt{\frac{ln(1/P_{fa})}{N_{w}}}\cdot (1-\gamma \cdot C(x,y))$$

where α=4, γ∈[0,1] is the structural adjustment factor, and $P_{fa}=10^{-4}$ is the false alarm rate.

If the following condition is satisfied, mark it as a target; otherwise, mark it as background.

(15) If X(x,y)>T(x,y)

Compared with traditional CFAR, this method places greater emphasis on the stability and continuity of the local structure, which helps improve the detection rate of weak targets, especially when the point cloud is sparse.

### 3.2. TPS-DWT

A large amount of random high-frequency interference in the Range–Doppler (RD) map originates from background thermal noise, non-ideal circuitry, and external reflection disturbances. These noises are predominantly distributed in the high-frequency subbands and exhibit characteristics such as non-structural randomness, local confinement, and small amplitude. Conventional wavelet denoising methods that apply a uniform soft threshold often suppress the target edge signals undesirably.

To address this issue, we propose a target-preserving sparse wavelet denoising strategy (TPS-DWT), which integrates multi-scale transformation, local energy estimation, and target fidelity control. This approach effectively suppresses noise while preserving essential structural details.

The input image X is decomposed using a three-level two-dimensional discrete wavelet transform (DWT):

(16) $$X\overset{DWT}{\to }{\left\{A_{3},D_{j}^{H},D_{j}^{V},D_{j}^{D}\right\}}_{j=1}^{3}$$

For each high-frequency coefficient $D_{j}(x,y)$, a structure-preserving soft threshold is constructed as

(17) $$\lambda_{j}(x,y)=\eta \cdot \hat{\sigma }\cdot \left(1-\frac{E_{\Omega }(x,y)}{E_{max}+\epsilon }\right)$$

where $\hat{\sigma }$ is the noise standard deviation estimated via the median absolute deviation (MAD) method, $E_{\Omega }(x,y)$ denotes the local energy measure defined as the sum of squared coefficients within a neighborhood, η is a scaling factor, and $E_{\max}$ is the maximum local energy at the current scale.

This strategy adaptively suppresses disturbances in noise-dominated regions while attenuating compression in target areas, thereby preserving target fidelity. The high-frequency coefficients are then processed by soft-thresholding:

(18) $${\hat{D}}_{j}(x,y)=sign(D_{j}(x,y))\cdot max(|D_{j}(x,y)|-\lambda_{j}(x,y),0)$$

Finally, the denoised image is reconstructed via the inverse DWT (IDWT):

(19) $$\hat{X}=IDWT\left(A_{3},{\hat{D}}_{1}^{*},{\hat{D}}_{2}^{*},{\hat{D}}_{3}^{*}\right)$$

**Algorithm 2:** Target-Preserving Sparse DWT (TPS-DWT)**Input:** RD map $X\in R^{M\times N}$; wavelet basis ψ; decomposition level L; **Output:** Enhanced RD map $\hat{X}$.
  1:perform L-level 2D Discrete Wavelet Transform:  2:

$\{A_{L},D_{L}^{H},D_{L}^{V},D_{L}^{D},\ldots ,D_{1}^{H},D_{1}^{V},D_{1}^{D}\}\leftarrow DWT(X,\psi );$

  3:**for** l = 1 to L **do**   4:      **for** each coefficient c in $\left\{D_{l}^{H},D_{l}^{V},D_{l}^{D}\right\}$ do  5:             Compute soft threshold;  6:             $c^{\prime }\leftarrow$ sign (c)⋅max(|c|−λ,0);  7:         **if** M indicates target region at coefficient c **then**   8:               Keep original coefficient;  9:               $\;\;\;\;\;\;\;c^{\prime }\leftarrow c$;  10:         **end**  11:         Update detail coefficient with $c^{\prime }$;  12:      **end**  13:**end**   14:Reconstruct enhanced RD map:   15:$\hat{X}\leftarrow IDWT\left(A_{L},D_{L}^{H^{\prime }},D_{L}^{V^{\prime }},D_{L}^{D^{\prime }},\ldots \right)$;  16:
return $\hat{X}$



It should be emphasized that the preprocessing module performs deterministic and non-learnable operations, such as normalization and basic signal conditioning, with the primary goal of stabilizing the input data distribution and improving training convergence. These operations do not aim to explicitly suppress complex interference patterns or enhance target responses.

## 4. Proposed Model

The preprocessing module and IM-Net serve complementary but fundamentally different roles within the proposed framework. While preprocessing provides coarse signal conditioning through deterministic operations, IM-Net is responsible for learning complex and non-linear interference characteristics that cannot be effectively addressed by fixed preprocessing techniques alone. This hierarchical design avoids functional redundancy and ensures that learning capacity is reserved for modeling interference patterns with high variability.

In this section, we introduce the proposed two-stage model for automotive radar interference mitigation. As illustrated in Figure 2, the proposed framework comprises two neural networks: the Interference Mitigation Network (IM-Net) and the Target Enhancement Network (TE-Net). The IM-Net is trained independently, while the TE-Net can be trained either separately or jointly with a pre-trained IM-Net. By collecting both clean and interference-contaminated signals in real-world scenarios, we propose a training strategy based on a real dataset containing discrete interference beat signals.

To effectively enhance the anti-interference capability and target discernibility of millimeter-wave FMCW radar under complex interference conditions, we design a deep generative adversarial network (GAN)-based architecture for interference suppression and target enhancement. This architecture consists of two primary modules: the IM-Net for suppressing interference and restoring clean Range–Doppler (RD) maps, and the TE-Net for further amplifying weak target reflections, thereby improving detection sensitivity.

The proposed RIME-Net adopts a two-stage architecture based on a task-decoupling design philosophy rather than a simple cascaded enhancement scheme. Specifically, IM-Net and TE-Net are designed to address two fundamentally different signal characteristics in the Range–Doppler (RD) domain.

IM-Net focuses on suppressing structured and globally distributed interference by exploiting its low-rank and broadband characteristics, which are difficult to eliminate using local enhancement strategies. In contrast, TE-Net is dedicated to enhancing sparse and localized target responses after interference suppression. By explicitly separating interference mitigation and target enhancement into two specialized stages, the proposed framework avoids jointly optimizing conflicting objectives within a single network, thereby reducing the risk of error accumulation and performance instability.

Accordingly, the proposed network does not rely on a strict global sparsity assumption of the RD map. Instead, it leverages the relative structural differences between target responses and interference patterns. The network focuses on enhancing locally salient and physically plausible target structures, rather than assuming that target responses are globally sparse across the entire RD domain.

### 4.1. Physics-Guided Unpaired Learning

The first stage of the proposed framework is architecturally designed to approximate the low-rank shrinkage operator defined in the structural decomposition model. As established in the conceptual framework, automotive radar interference—such as broadband noise and ghost targets—manifests as structured, globally distributed energy patterns that can be modeled as a low-rank manifold within the range–Doppler (RD) space. To effectively isolate this manifold without the need for elusive paired training samples, the framework utilizes a Cycle-Consistent Adversarial architecture to learn the domain translation between the interference-contaminated domain and the clean domain. This choice is physically motivated by the practical constraints of real-world radar acquisition, where simultaneous clean and interfered measurements under identical environmental conditions are unattainable. The framework employs dual generators and PatchGAN-based discriminators to ensure that the statistical distribution of the restored RD map strictly aligns with interference-free radar physics. To preserve the point-like scattering features of radar targets, the generator adopts a U-Net backbone with symmetric skip connections. While the encoder phase progressively extracts high-level semantic features and suppresses stochastic noise via strided convolutions, the skip connections function as identity pathways that reintroduce low-level spatial details back to the decoder, thereby preventing the “over-smoothing” of target edges.

The “physics-guided” nature of this unpaired learning stage is further enforced by a composite objective function that constrains the network optimization within physically plausible bounds. Specifically, a Cycle-Consistency Loss is applied to ensure the conservation of signal information, requiring that the signal translated to the clean domain can be reconstructed back to its original state. To maintain the frequency-domain integrity and phase-coupling characteristics inherent in FMCW signal processing, we introduce a Spectral Consistency Loss ($L_{\mathrm{spec}\;}$), which is formulated as

(20) $$L_{\mathrm{spec}\;}=E_{x}∥F(x)-F(F(G(x))){∥}_{1}+E_{y}∥F(y)-F(G(F(y))){∥}_{1}$$

where F(•)  denotes the 2D Fourier transform. This loss term effectively forces the generator to respect the original spectral signatures of the radar echoes. Additionally, to preserve spatial structural similarity and reduce artifacts, a structural similarity index measure (SSIM) loss is used:

(21) $$L_{s\mathrm{sim}\;}=1-SSIM\left(x,F\left(G\left(x\right)\right)\right)+1-\mathrm{SSIM}(y,G(F(y)))$$

By integrating these structural and functional constraints, this stage transcends the limitations of “black-box” image processing, functioning as a learnable surrogate for traditional matrix decomposition in complex electromagnetic environments.

### 4.2. IM-Net

The Interference Suppression Network is constructed based on the CycleGAN framework, which provides strong unsupervised learning capability and is well-suited for training with unpaired clean and interference-contaminated RD maps in this study. Specifically, the ISN module consists of two generators and two discriminators, which learn the mappings from interference-contaminated RD maps to clean RD maps and the inverse mapping, respectively. To ensure that the content of the original image is preserved after translation, CycleGAN introduces a cycle-consistency loss. By combining adversarial loss and cycle-consistency loss, the ISN module can learn the mapping from contaminated to clean RD maps without paired data, thereby achieving radar interference suppression.

**Figure 3:**
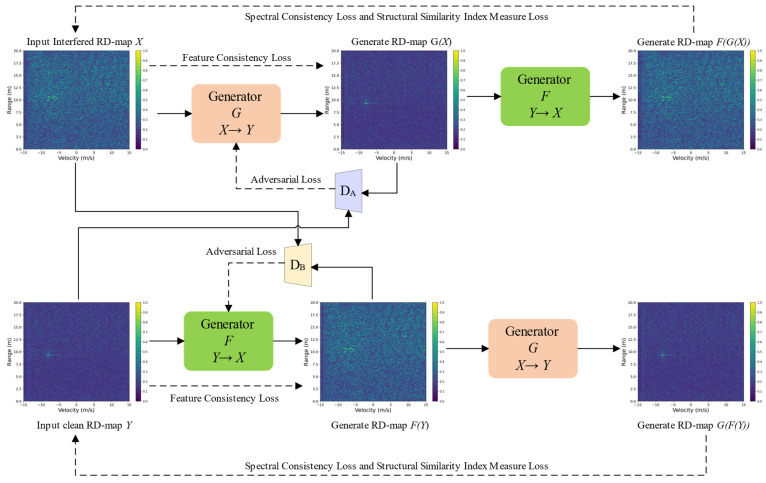
Network architecture of the IM-Net module. The dashed line represents the backpropagation process.

Furthermore, to enhance model performance, identity loss and perceptual loss are incorporated. The identity loss helps maintain the color composition between input and output images, preventing color shifts, while the perceptual loss enhances structural and textural details by comparing high-level feature representations. Through these strategies, the IM-Net extracts clean features from interference-contaminated RD maps, improving radar system performance under complex interference conditions. The specific structure of IM-NET is shown in Figure 3.

In this study, to effectively recover clean radar signals from interference-contaminated RD images, we design and implement a generator network based on the U-Net architecture. The network has a symmetric encoder–decoder structure with skip connections, which directly pass feature maps from the encoder to the decoder to preserve spatial details, particularly the scattering signal details of radar targets.

The encoder consists of several convolutional layers that progressively extract high-level features from the input image while reducing the spatial resolution of the feature maps. Each convolutional layer uses a kernel size of 4 × 4 with a stride of 2. LeakyReLU activations are employed throughout, and batch normalization is applied to intermediate layers to accelerate convergence and improve stability.

The decoder mirrors the encoder with transposed convolutional layers that gradually restore the spatial resolution of the feature maps. ReLU activations and batch normalization are applied to all intermediate layers, while the final layer uses a Tanh activation to normalize the output to the range [−1, 1]. Skip connections between the encoder and decoder enable the network to leverage low-level features extracted by the encoder, allowing the decoder to effectively reconstruct fine spatial details. Mathematically, if x denotes the input image, the outputs of the encoder and decoder layers are combined via concatenation, enhancing the preservation of spatial detail during reconstruction.

**Figure 4:**
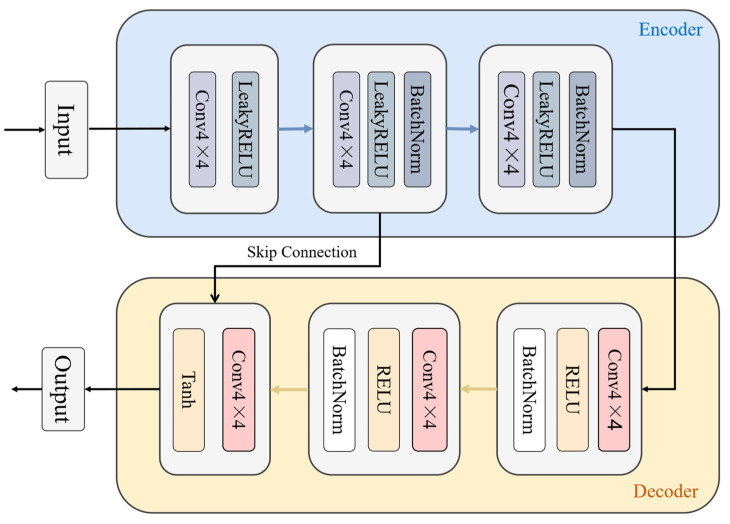
Architecture of the generator network.

Through the specific design shown in Figure 4, the generator network can effectively extract clean radar signals from interference-contaminated RD images while maintaining target details, thereby improving the performance and reliability of the radar system under complex environments.

To evaluate the quality of RD images generated by the generator, a PatchGAN discriminator is employed. PatchGAN divides the input image into multiple patches and performs real/fake discrimination on each patch, rather than on the entire image. This local discrimination emphasizes structural details and high-frequency information, which is crucial for detecting and reconstructing small targets in radar images.

**Figure 5:**
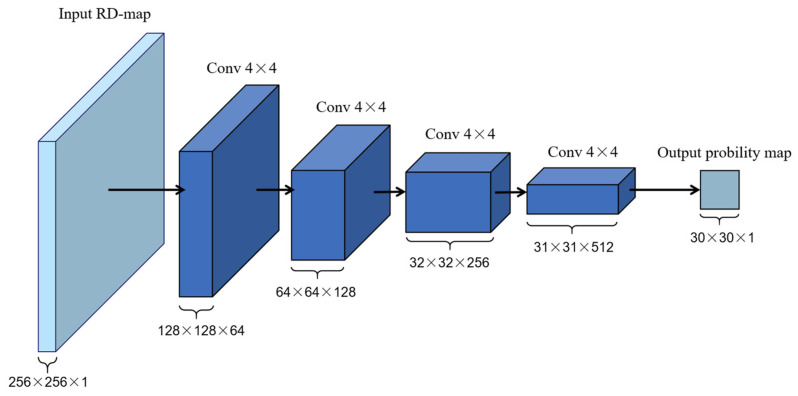
Architecture of the discriminator network.

The discriminator consists of multiple convolutional layers with LeakyReLU activations to introduce nonlinearity and prevent gradient vanishing, progressively reducing the spatial resolution and producing a probability map for each patch. The final output is obtained by averaging these probabilities. This architecture improves sensitivity to local differences and encourages the generator to produce images with accurate details, enhancing the overall quality and realism of the reconstructed RD images. The PatchGAN-based discriminator effectively captures and evaluates local details in radar images. The structure of the discriminator is shown in Figure 5.

To ensure that the generated RD maps are indistinguishable from real samples in both the interfered domain X and the clean domain Y, adversarial losses are imposed on the generators G:X→Y and F:Y→X.

For discriminator $D_{Y}$, which distinguishes real clean RD maps from generated ones, the adversarial loss is formulated as

(22) $$L_{\mathrm{adv}}^{G}=E_{y∼Y}\;[logD_{Y}(y)]+E_{x∼X}\;[log(1-D_{Y}(G(x)))]$$

Similarly, for discriminator $D_{X}$ operating on the interfered domain:

(23) $$L_{\mathrm{adv}}^{F}=E_{x∼X}\;[logD_{X}(x)]+E_{y∼Y}\;[log(1-D_{X}(F(y)))]$$

The adversarial losses guide the generators to produce realistic RD maps with correct global statistical structures.

To preserve the structural information and avoid distortion during the domain translation processes X↔Y, a feature consistency loss is applied.

Given a shared feature extractor ϕ(⋅), the loss enforces similarity between the input RD map and its reconstructed counterpart:

(24) $$L_{\mathrm{feat}}={∥\phi (x)-\phi (F(G(x)))∥}_{1}+{∥\phi (y)-\phi (G(F(y)))∥}_{1}$$

This term preserves semantic and structural cues such as target shape, local scattering patterns, and interference morphology.

To ensure that the translated RD maps retain correct spectral signatures and remain consistent with radar physical characteristics, a spectral consistency term is introduced.

Let F(⋅) denote the 2D Fourier transform. The spectral consistency loss is

(25) $$L_{\mathrm{spec}}={∥F(x)-F(F(G(x)))∥}_{1}+{∥F(y)-F(G(F(y)))∥}_{1}$$

Additionally, to preserve spatial structural similarity and reduce artifacts, a structural similarity index measure (SSIM) loss is used:

(26) $$L_{\mathrm{ssim}}=1-\mathrm{SSIM}(x,F(G(x)))+1-\mathrm{SSIM}(y,G(F(y)))$$

The combination of spectral and SSIM constraints maintains both frequency-domain integrity and spatial structural fidelity, which are essential for accurate radar target representation.

### 4.3. TE-Net

The proposed two-stage framework adopts a hierarchical and cooperative processing strategy between IM-Net and TE-Net. Specifically, IM-Net is designed to suppress large-scale and structured interference components while preserving the essential target-related structures in the Range–Doppler domain. The output of IM-Net therefore serves not only as an interference-mitigated RD representation, but also as a structural prior that highlights potential target regions and suppresses residual interference.

Based on this intermediate representation, TE-Net performs a constrained target enhancement process. Instead of blindly amplifying all signal components, TE-Net leverages the IM-Net output as feature guidance, focusing the enhancement on regions with high target confidence while avoiding the amplification of residual artifacts. This cooperative mechanism enables hierarchical processing, where interference suppression and target enhancement are decoupled yet mutually constrained, effectively reducing information loss and improving robustness in complex multi-target scenarios.

Following the interference mitigation stage, weak targets in the range–Doppler (RD) map may still suffer from low amplitude, blurred contours, or incomplete structural information. To address these issues, we design a lightweight and structure-aware Target Enhancement Network (TE-Net), which serves as the second stage of the proposed two-stage restoration framework. TE-Net adopts an encoder–decoder architecture and integrates multi-level residual enhancement and channel attention mechanisms to selectively amplify target responses while avoiding the unintended reinforcement of background noise.

The design of the target enhancement network (TE-Net) is closely related to the sparse–low-rank joint modeling described in Section 2.3. From a physical modeling perspective, the interference-mitigated Range–Doppler (RD) map can be expressed as the superposition of sparse target responses and residual structured components, which can be written as

(27) Y=S+R

where Y denotes the RD map output by IM-Net, S represents the sparse target response, and R corresponds to residual interference and background components that typically exhibit low-rank or structured characteristics. Based on this model, the ideal target enhancement process can be formulated as a sparse–low-rank decomposition problem, expressed as

(28) $$\underset{S}{min}∥S{∥}_{0}+\;\lambda ∥Y-S\;{∥}_{*}$$

where $∥•{∥}_{>0}$ enforces sparsity on the target response and $∥•{∥}_{*}$ denotes the nuclear norm that promotes low-rank structure in the residual components. Although this formulation provides a physically meaningful interpretation of target enhancement in the RD domain, explicitly solving such an optimization problem usually requires iterative procedures and incurs high computational cost, making it unsuitable for real-time automotive radar applications. Instead, TE-Net is designed to implicitly approximate this physics-consistent mapping through a learnable nonlinear function, which can be expressed as

(29) $$\hat{S}=F_{TE}(Y;\theta )$$

where $\hat{S}$ denotes the enhanced target response and θ represents the network parameters. From this perspective, TE-Net can be interpreted as a data-driven surrogate for the sparse–low-rank decomposition process. The convolutional layers facilitate local modeling of sparse target structures in the RD domain, while the attention mechanism adaptively emphasizes physically meaningful target features and suppresses residual low-rank artifacts. In addition, residual connections are employed to preserve weak target information and avoid over-smoothing, which is consistent with the physical characteristics of weak radar echoes. Through this design, radar physical priors guide the learning process in an implicit yet effective manner, without explicitly embedding physical equations or performing iterative optimization.

Unlike conventional enhancement networks that perform unconstrained feature amplification, TE-Net is designed as a constrained residual enhancement module. Specifically, the network learns a residual refinement over the output of IM-Net instead of regenerating a new RD representation from scratch. This residual formulation inherently limits the magnitude of enhancement and prevents the creation of artificial structures.

Furthermore, TE-Net incorporates channel–spatial attention mechanisms to adaptively modulate feature responses. These mechanisms suppress noise-dominant and interference-like components while selectively emphasizing sparse and target-relevant features that exhibit physically plausible localization in the RD domain. As a result, residual artifacts potentially remaining after IM-Net are more likely to be attenuated than amplified.

**Figure 6:**
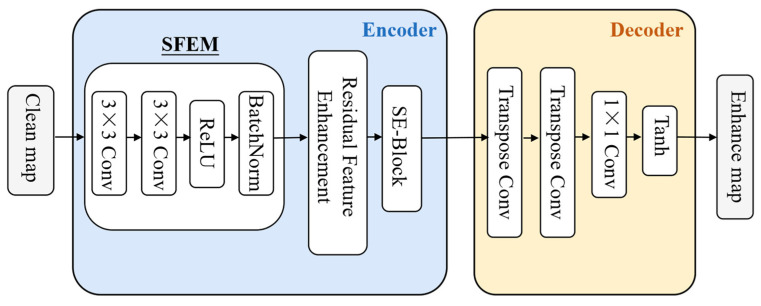
Network architecture of the TE-Net module.

The overall architecture of TE-Net is shown in Figure 6. It is composed of three major components:

1. A Shallow Feature Extraction Module (SFEM);
2. An encoder with residual and attention-based enhancement;
3. A decoder for spatial reconstruction and amplitude normalization.

The SFEM aims to capture basic local textures and edge information from the clean RD map. It consists of two consecutive 3 × 3 convolution layers, followed by ReLU activation and Batch Normalization:

(30) $$F_{1}=\mathrm{BN}(\mathrm{ReLU}({\mathrm{Conv}}_{3\times 3}({\mathrm{Conv}}_{3\times 3}(X))))$$

where X denotes the input clean RD map. This module increases the network sensitivity to fine-scale target boundaries and reduces irrelevant background fluctuations.

To improve the model’s ability to extract high-level semantic and structural information, multiple residual enhancement blocks are stacked in the encoder. Each residual block consists of two 3 × 3 convolution layers with ReLU activation and a skip connection:

(31) $$R_{i}=F_{i-1}+{\mathrm{Conv}}_{3\times 3}(\mathrm{ReLU}({\mathrm{Conv}}_{3\times 3}(F_{i-1})))$$

These residual pathways stabilize the gradient flow during training, increase feature abstraction capability, and enhance multi-scale responses of weak targets.

To selectively emphasize target-related channels and suppress noise-dominant channels, a Squeeze-and-Excitation (SE) block is integrated after the residual enhancement module. The SE-block performs global feature aggregation followed by channel-wise reweighting:

(32) $$F_{\mathrm{att}}=F\cdot \sigma (W_{2}\;\mathrm{ReLU}(W_{1}\;\mathrm{GAP}(F)))$$

where GAP denotes global average pooling, $W_{1}$ and $W_{2}$ are learnable parameters, and σ(⋅) is the sigmoid function. The attention mechanism forces the network to focus on high-energy, target-correlated channels.

The decoder consists of two transposed convolution layers to gradually restore spatial resolution and reconstruct enhanced target signatures. A 1 × 1 convolution is then applied to reduce the channel dimension to one, matching the RD map format. The final activation function is Tanh, which normalizes the output into [−1,1]

(33) $$Y=tanh({\mathrm{Conv}}_{1\times 1}(\mathrm{TConv}(\mathrm{TConv}(F_{\mathrm{att}}))))$$

The decoder strengthens spatial continuity, restores fine-scale structure, and outputs the enhanced RD map with preserved physical constraints.

## 5. Experiment

### 5.1. Experimental Setup and Dataset

**Figure 7:**
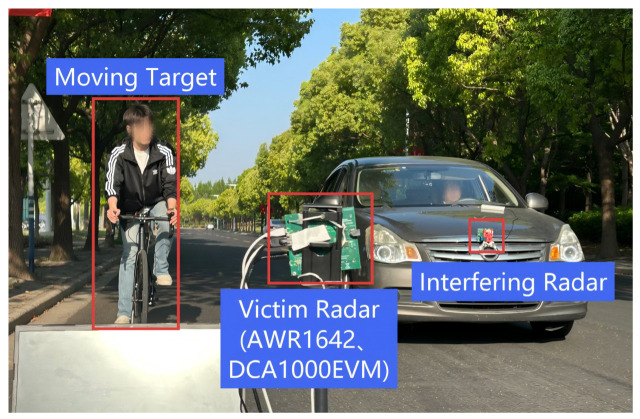
Experimental radar data acquisition setup.

To comprehensively evaluate the performance and robustness of the proposed two-stage radar interference mitigation and target enhancement framework (RIME-Net), we construct multiple representative experimental scenarios using both simulated and real-world data collected from an in-house automotive FMCW radar platform. All experiments are conducted on normalized range–Doppler (RD) maps with a spatial resolution of 128 × 128, and the output of the model is the enhanced RD map after interference suppression and target refinement. The experimental scenario we designed in the real world is shown in Figure 7.

In this work, the “clean” Range–Doppler (RD) maps are obtained independently from the interference-contaminated data. Specifically, clean data are collected under interference-free conditions, where no external radar interference sources are present, while using the same radar configuration and signal processing chain. In addition, simulated clean RD maps are generated based on standard FMCW radar signal models without injecting interference components.

All real-world measurements were acquired using a self-developed Texas Instruments AWR1642 mmWave radar. The Texas Instruments AWR1642 mmWave radar was manufactured by Texas Instruments, headquartered in Dallas, TX, United States. Table 1 summarizes the key system parameters for the signal-acquisition radar and the interference transmitter. For real data acquisition, the interference intensity is controlled by adjusting the relative transmission power of the interference source or the relative distance between the interfering radar and the victim radar. By varying these parameters, different interference power levels can be obtained in a controlled manner. Target distances are determined based on known geometric configurations of the experimental setup, where targets are placed at predefined ranges and calibrated using standard radar ranging procedures.

These configurations ensure that the dataset contains diverse interference patterns, including beat-frequency collisions, slope-mismatch interference, and multi-path reflections.

To generate controlled and repeatable test sets, we additionally construct a set of parametric radar scenes. Table 2 lists the parameter ranges used for target and interference simulation.

The interference chirp slope factor in Table 2 is defined as the ratio between the chirp slope of the interfering radar and that of the victim radar. This factor directly influences the manifestation of interference in the Range–Doppler domain, determining whether the interference appears as concentrated artifacts or distributed stripe-like patterns. By varying the chirp slope factor, different realistic interference behaviors observed in automotive radar systems can be effectively modeled.

For simulated data, interference patterns are generated based on standard FMCW radar signal models. Specifically, interference signals are synthesized by introducing asynchronous chirps with varying slopes, starting frequencies, and time offsets relative to the victim radar. These simulated interference patterns are designed to closely resemble real-world automotive radar interference scenarios, including both narrowband and wideband interference effects observed in practical measurements.

These configurations allow the construction of a wide range of RD scenes, including weak targets, high-power interference, and multi-target cluttered environments.

To assess generalization across different operational conditions, three representative categories of scenarios are designed:

- Mild interference scenes—low-intensity cross-radar interference with moderate SNR.
- Severe interference scenes—strong ridge-shaped, patch-type and broadband interference significantly corrupting the RD spectrum.
- Multi-target/weak-target scenes—distant targets, low-RCS objects, and dense multi-object environments.

All real-world experiments adopt the same data pipeline: raw ADC signals → 2D-FFT → RD map → preprocessing (SC-CFAR + TPS-DWT) → network inference.

For all experiments, the datasets are divided into training, validation, and test sets following a consistent and fair evaluation protocol. Specifically, the simulated dataset is randomly split into 70% for training, 15% for validation, and 15% for testing. To avoid data leakage, samples belonging to the same simulated scenario are assigned exclusively to one subset.

For the real-world automotive radar dataset, the data are split at the sequence and scene level rather than at the individual frame level. Approximately 70% of the collected driving sequences are used for training, 10% for validation, and the remaining 20% for testing. This ensures that testing data are acquired from different driving scenarios and interference conditions that are not observed during training. All models are trained using the same data splits to guarantee a fair comparison, and the reported quantitative results are obtained exclusively from the test sets.

All models are trained on a high-performance computing workstation equipped with two NVIDIA RTX 3090 GPUs, using PyTorch 2.0 and CUDA 11.7. The Adam optimizer is used with parameters β_1_ = 0.5 and β_2_ = 0.999. The initial learning rate is set to 2 × 10^−4^, kept constant for the first 50 epochs, and linearly decayed for the remaining 50 epochs.

IM-Net (Stage 1) is trained for 100 epochs to ensure sufficient learning of interference-to-clean domain mapping.

TE-Net (Stage 2) is subsequently trained for 50 epochs, using the outputs of IM-Net as input to refine weak targets.

The batch size is set to 32, enabling stable gradient updates for high-resolution RD data. To further enhance the sensitivity to target regions, a target-aware mask is incorporated during training to guide the enhancement network toward preserving and amplifying true target structures.

With this comprehensive experimental configuration, the proposed RIME-Net is systematically validated across diverse interference conditions and target distributions, ensuring a fair and thorough evaluation of its interference mitigation and target enhancement capabilities.

### 5.2. Preprocessing Results

**Figure 8:**
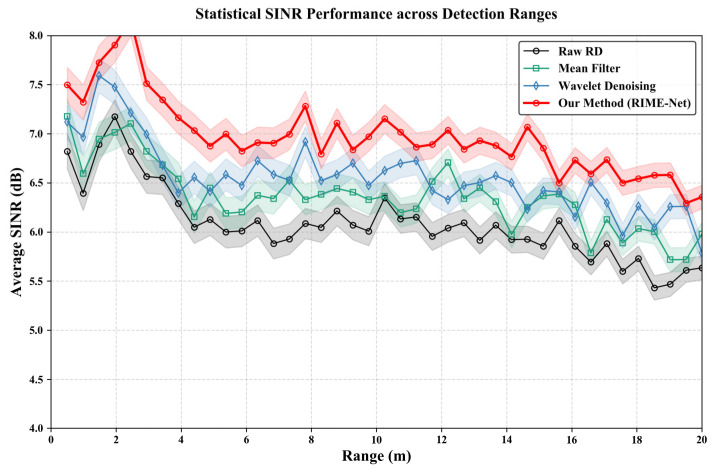
SINR comparison among different denoising methods.

To validate the effectiveness of the proposed preprocessing pipeline, including the Structure-Consistent CFAR (SC-CFAR) detector and the TPS-DWT–based interference suppression module, we apply both methods to the raw RD maps prior to network inference. Figure 8 illustrates the average SINR improvement across different target ranges (0–20 m), comparing the raw RD maps with several classical preprocessing baselines, including the mean filter and wavelet-based denoising.

As shown in Figure 7, the raw RD data exhibit a relatively low and unstable SINR, especially in mid-range regions where background clutter and residual interference are prominent. Traditional mean filtering provides limited enhancement due to its inability to preserve localized target structures, while wavelet denoising achieves moderate improvement but still suffers from oversmoothing in low-SNR regions.

In contrast, the proposed preprocessing module achieves consistently superior SINR across the entire range domain. The TPS-DWT effectively eliminates broadband interference while retaining fine-grained structural information, and the SC-CFAR further suppresses clutter by adaptively leveraging local structural consistency. Together, these operations yield a significant SINR gain of approximately 0.5–1 dB compared with conventional denoising methods, while maintaining stable performance even for distant targets (15–20 m).

These results demonstrate that the proposed preprocessing pipeline provides robust interference suppression and enhances the quality of RD maps, laying a solid foundation for the subsequent two-stage deep network to extract more discriminative features and improve target enhancement performance.

As illustrated in Figure 8, the proposed RIME-Net achieves a consistent SINR gain of approximately 0.5–1.0 dB over traditional Wavelet Denoising and Mean Filtering across the range of 0–20 m. While seemingly marginal in logarithmic scale, this improvement is of paramount importance for edge-case detection.

As shown in the updated Figure 8, we have incorporated shaded regions representing ±1 standard deviation to account for the stochastic nature of radar returns. While the raw SINR exhibits inherent oscillatory behavior due to the range-dependent path loss and complex multi-path reflections in urban environments, our proposed SC-CFAR + TPS-DWT pipeline yields a statistically significant improvement of approximately 0.5–1.0 dB over standard Mean Filtering and Wavelet Denoising. This gain is consistent across the 0–20 m range, demonstrating that our structural-consistency-aware approach effectively stabilizes the RD-map distribution before it enters the high-capacity IM-Net.

**Figure 9:**
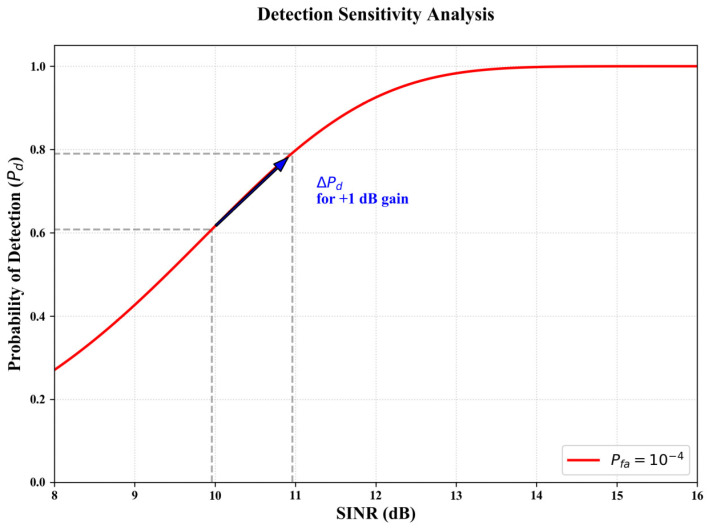
Detection sensitivity analysis. The dashed lines indicate the corresponding SINR and detection probability values before and after a 1 dB gain, while the blue arrow denotes the resulting increase in detection probability.

To quantitatively justify the significance of the SINR improvements, we further provide a detection sensitivity analysis (Figure 9). In radar detection theory, the probability of detection ($P_{d}$) is a highly non-linear function of SINR, particularly in the critical transition region. As demonstrated, a marginal gain of 1.0 dB near the detection threshold (e.g., around 12 dB) can elevate the $P_{d}$ from 0.61 to 0.82. This enhancement effectively mitigates missed detections for low-RCS targets, explaining the substantial improvements in Average Precision (AP) despite the relatively low logarithmic gains in average SINR.

According to the radar detection sensitivity model, the transition from ‘missed detection’ to ‘successful acquisition’ often occurs within a narrow SINR window. A 1 dB gain at the detection threshold can significantly steepen the $\;P_{d}$ curve, ensuring that weak reflectors (e.g., pedestrians at 15 m) are reliably identified rather than submerged in the noise floor. Furthermore, the stability of the ‘Our method’ curve in Figure 7, compared to the oscillatory behavior of raw RD data and standard filters, demonstrates the robustness of the learned physical priors. The efficacy of this gain is further corroborated by the systemic metrics in Section 5.3, where RIME-Net demonstrates a 4.14% and 6.46% higher AP than ResNet and End-to-End Net, respectively, under severe interference scenarios.

### 5.3. Mild Interference Scenario

Building upon the preliminary denoising benchmarks established in Section 5.2—where the fundamental limitations of traditional filters were quantitatively and qualitatively addressed—this section evaluates the advanced interference mitigation performance of RIME-Net against other deep learning frameworks. While the ‘raw’ state and basic denoising performance are documented in Section 5.2 and Figure 7, the following visual comparisons in Figure 8 focus on the structural fidelity achieved by high-capacity neural architectures.

Light interference typically occurs in non-line-of-sight (NLOS) radar interactions at relatively long distances, where the interfering stripes remain sparse and of low amplitude, and the target signatures are still clearly distinguishable. Such interference is common in multi-vehicle environments in which mutual radar emissions only weakly overlap in time-frequency space. Although the disturbance does not dominate the RD map, it still introduces noticeable fluctuations in the background floor and may cause partial blurring or local distortion of weak target responses.

The objective of this experimental setting is to evaluate the model’s capability in suppressing mild interference while preserving the inherent structural properties of the RD map. Since the target regions are still visually recognizable under light interference, the primary challenge lies not in reconstructing missing features but in avoiding excessive smoothing, artifact generation, or deformation of the fine-grained target signatures. Therefore, this scenario is particularly suitable for examining the balance between interference removal and structural fidelity. It also provides a baseline for comparing how different algorithms handle subtle background disturbances without degrading target clarity.

**Figure 10:**
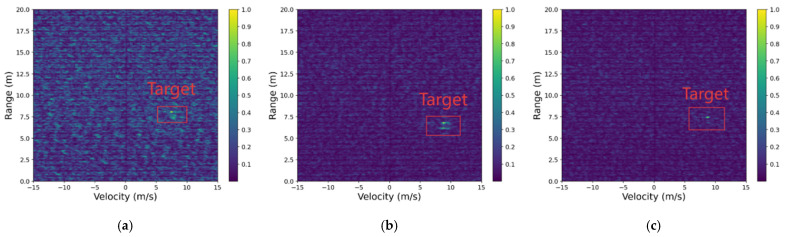

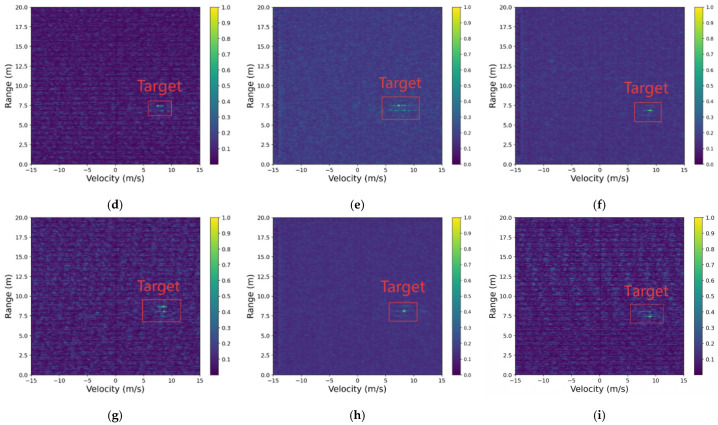
RD map comparison of various models under mild interference. (**a**) Raw interference Contaminated RD map before processing. (**b**) ResNet. (**c**) DnCNN. (**d**) U-Net. (**e**) Autoencoder. (**f**) Radar-STDA. (**g**) End-to-End Net. (**h**) RIME-Net. (**i**) RD map after preprocessing.

The following Figure 10 illustrates the denoising and enhancement performance of each model on RD maps under mild interference conditions. The content enclosed by the red box corresponds to the target signal that we aim to recover. Visually, DnCNN, U-Net, End-to-End, and ResNet can generally recover the main target contours, but exhibit varying degrees of texture loss and background blurring; Autoencoder and U-Net produce diffused target contours after interference suppression, making the targets less distinguishable. The output of RIME-Net shows significantly better interference suppression than traditional methods and preserves more structural details. Furthermore, after enhancement by EnhanceNet, the target contrast is markedly improved, and the edge regions become clearer.

To comprehensively assess the effectiveness of RIME-Net, we employ five complementary metrics to evaluate both reconstruction quality and detection performance.

Signal-to-Interference-plus-Noise Ratio (SINR) measures the ratio of the target signal power to the sum of interference and background noise power, characterizing the enhancement of target visibility:

(34) $$SINR=10{log}_{10}\left(\frac{P_{signal}}{P_{interference}+P_{noise}}\right)$$

Mean Squared Error (MSE) quantifies the pixel-wise intensity deviation between the restored RD-map $\hat{X}$ and the clean reference X:

(35) $$\mathrm{MSE}=\frac{1}{MN}\sum_{i=1}^{M}\sum_{j=1}^{N}(X_{i,j}-{\hat{X}}_{i,j}{)}^{2}$$

Structural Similarity (SSIM) assesses the preservation of spatial patterns and structural integrity (luminance, contrast, and structure) in the RD-map.

Average Precision (AP) evaluates the target recovery capability by calculating the area under the Precision-Recall (PR) curve, reflecting the trade-off between sensitivity and precision.

The False Positive Rate (FPR) represents the probability of erroneously identifying interference artifacts as true targets:

(36) $$\mathrm{FPR}=\frac{FP}{FP+TN}$$

where FP and TN denote false positives and true negatives, respectively. The inclusion of both regression-based metrics (MSE, SSIM) and classification-based metrics (AP, FPR) is critical for automotive radar. While MSE measures global fidelity, FPR specifically monitors the suppression of misleading artifacts, ensuring the system satisfies the stringent safety requirements of autonomous driving.

Table 3 summarizes the quantitative results of all competing models across five commonly used metrics: SINR, MSE, AP, FPR, and SSIM. The results show clear performance differences among traditional denoising networks, general-purpose deep models, and the proposed RIME-Net.

Overall, RIME-Net achieves the best performance across all metrics. Specifically, it obtains the highest SINR (23.75 dB), indicating its strong capability in suppressing interference while preserving signal energy. It should be emphasized that the reported SINR value of 23.75 dB is calculated relative to the original interfered Range–Doppler (RD) map. All comparative methods are evaluated under the same interfered input condition, which guarantees fairness and consistency in the quantitative performance assessment. It also yields the lowest MSE (0.0084), showing lower reconstruction error compared with the other methods. In terms of detection performance, RIME-Net achieves the highest AP (90.45%) and the lowest FPR (0.0375), demonstrating more accurate target recovery and fewer false alarms. Furthermore, it reaches the highest SSIM (0.948), suggesting superior preservation of structural information in the restored RD maps.

Among the baseline models, ResNet and End-to-End Net perform relatively well, with SINR values of 20.62 dB and 19.54 dB, AP values of 86.31% and 85.73%, and SSIM scores of 0.912 and 0.923, respectively. However, both exhibit higher MSE and FPR compared with RIME-Net. Lightweight models such as DnCNN and U-Net show noticeably weaker performance, reflected by lower SINR (13.68 dB and 16.31 dB), higher MSE, and degraded structural similarity. Autoencoder and Radar-STDA perform moderately, showing improvements over basic CNN models but still falling behind the proposed network across all evaluation measures.

These comparisons demonstrate that RIME-Net consistently outperforms conventional denoising networks, encoder–decoder architectures, and existing radar-specific models, confirming its effectiveness in interference suppression, target reconstruction, and structural fidelity maintenance under challenging radar conditions.

### 5.4. Severe Interference Scenario

Following the hierarchical evaluation protocol, we further assess the robustness of RIME-Net under severe interference. As previously established in the preprocessing analysis (Section 5.2), standard linear denoising often fails to suppress high-energy structured artifacts. Consequently, the visual analysis in Figure 9 is dedicated to a SOTA-level comparison, emphasizing the decoupling capability of the proposed two-stage framework in extreme environments.

In dense urban traffic scenarios, automotive radars frequently encounter high-amplitude periodic interference—such as multi-radar echoes, near-field reflector returns, and power intermodulation. These interferences manifest in the RD map as large-area horizontal “stripes” or “blotches” that severely obscure targets. The objective of this experiment is to validate the proposed model’s interference suppression capability under extremely low SNR conditions.

**Figure 11:**
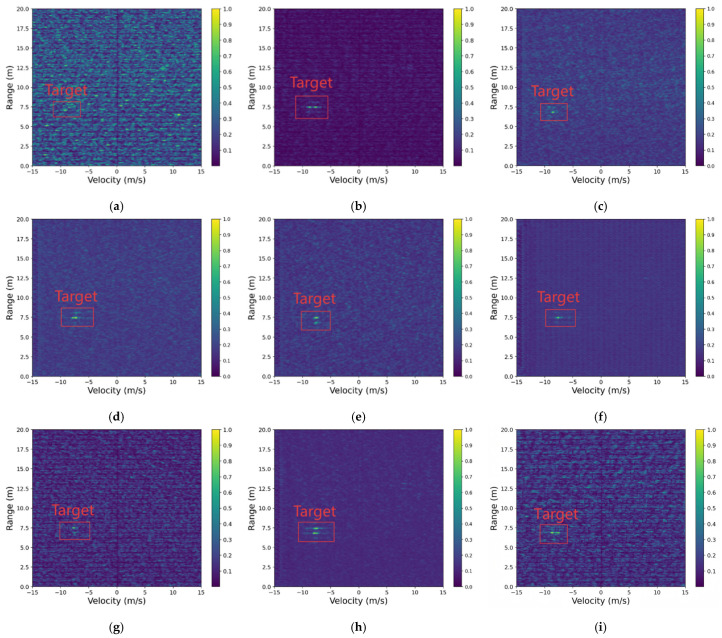
RD map comparison of various models under severe interference. (**a**) Raw interference Contaminated RD map before processing. (**b**) ResNet. (**c**) DnCNN. (**d**) U-Net. (**e**) Autoencoder. (**f**) Radar-STDA. (**g**) End-to-End Net. (**h**) RIME-Net. (**i**) RD map after preprocessing.

Figure 11 shows reconstruction results for a heavily interfered frame obtained by each method. The content enclosed by the red box corresponds to the target signal that we aim to recover. DnCNN completely fails to recognize the target region, producing an overall blurred output; although the U-Net output is somewhat clearer, it exhibits obvious artifacts and target misplacement. RIME-Net effectively suppresses the strong horizontal interference across the entire image and restores the weak central target well; the enhanced output has clear layering and virtually no redundant signals.

Table 4 summarizes the quantitative performance of seven representative denoising and reconstruction methods under a severe interference scenario, where the RD map is dominated by wideband, high-energy strip-like artifacts that severely obscure weak targets. As shown, traditional CNN-based denoisers such as DnCNN and U-Net exhibit significant performance degradation, with low SINR values (10.24 dB and 12.86 dB, respectively) and high MSE, indicating their limited ability to suppress strong structured interference. Their elevated FPR further suggests frequent misclassification of interference as targets.

The Autoencoder and Radar-STDA models provide slightly improved stability, yet still struggle to reconstruct weak targets, yielding only moderate AP values (74.83% and 76.21%). The End-to-End Net performs better than the aforementioned baselines, especially in terms of SSIM (0.885) and AP (78.70%), highlighting the benefits of unified representation learning.

In contrast, the proposed RIME-Net achieves the best performance across all metrics. It delivers the highest SINR (19.48 dB) and the lowest MSE (0.0112), demonstrating its strong capability to remove high-energy structured interference while preserving target information. Additionally, RIME-Net attains the highest AP (85.16%) and the lowest FPR (0.0547), confirming its robustness in accurately recovering weak targets without introducing spurious detections. Its SSIM score (0.918) further indicates superior structural fidelity in the reconstructed RD maps. These results collectively show that RIME-Net provides the most reliable reconstruction and interference suppression performance under severe interference conditions.

### 5.5. Multi-Target Scenario Analysis

**Figure 12:**
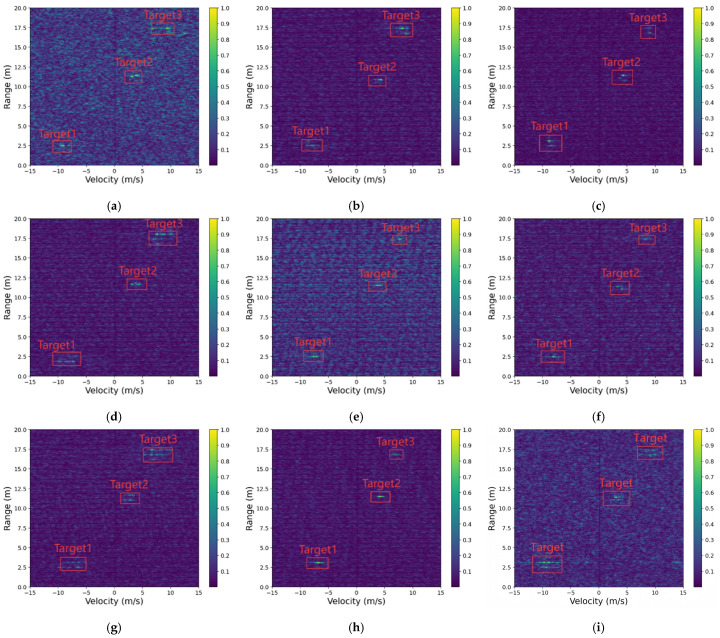
RD map comparison of various models in multi-target scenarios. (**a**) Raw interference Contaminated RD map before processing. (**b**) ResNet. (**c**) DnCNN. (**d**) U-Net. (**e**) Autoencoder. (**f**) Radar-STDA. (**g**) End-to-End Net. (**h**) RIME-Net. (**i**) RD map after preprocessing.

This scenario contains three real targets of different sizes and significantly varying energy levels. Among them, some targets correspond to distant weak reflectors with small radar cross-sections (RCS), making them highly susceptible to being submerged by background interference. The interference strength in this setting is moderate, with an original SINR of approximately 12–15 dB. To effectively evaluate the model’s capability under such challenging conditions, this scenario emphasizes simultaneous recovery of both strong and weak targets while maintaining structural fidelity. The coexistence of multiple targets with distinct reflectivity levels presents a more complex reconstruction problem, as weak targets are easily masked by clutter or residual interference, and strong targets may dominate the dynamic range of the RD map. Therefore, this scenario serves as a crucial benchmark for assessing the robustness of RIME-Net in multi-target environments, particularly its ability to enhance weak reflectors without compromising strong-target integrity. The specific comparison chart is shown in Figure 12. The content enclosed by the red box corresponds to the target signal that we aim to recover.

Table 5 reports the quantitative evaluation of seven representative models under the multi-target scenario, where the RD map contains three targets of different sizes and reflectivity levels. All baseline methods exhibit varying degrees of performance degradation in this more challenging setting. Traditional denoising networks such as DnCNN and Autoencoder suffer from higher MSE and reduced AP, indicating difficulty in simultaneously recovering multiple targets of unequal strength.

U-Net, Radar-STDA, and the End-to-End network achieve moderate improvements in SINR and SSIM, yet their FPR remains relatively high, reflecting limited capability in suppressing clutter while preserving weak targets. In contrast, the proposed RIME-Net consistently outperforms all comparison methods across all metrics, achieving the highest SINR (21.62 dB), best AP (88.73%), lowest MSE (0.0098), and lowest FPR (0.0451). These results demonstrate that RIME-Net can more effectively enhance multiple targets with different energy levels while maintaining accurate background suppression and structural fidelity.

It should be noted that all baseline methods selected for comparison are representative approaches proposed in recent years. To ensure fairness and consistency, the same set of baseline methods is evaluated across all three experimental scenarios considered in this work. By applying identical comparison methods under different interference and target configurations, the performance of RIME-Net can be systematically assessed under unified evaluation conditions.

### 5.6. Ablation Experiments and Efficiency Analysis

To evaluate the effectiveness of each key component in the proposed model, a systematic ablation study was conducted. All variants were trained and tested under the same dataset configuration, where different modules—namely the attention module (CBAM), the target enhancement module (TE-Net), and the interference mitigation module (IM-Net)—were progressively removed or added. By comparing the performance variations across these configurations, the individual contribution of each component can be quantitatively assessed.

Table 6 summarizes the results under the heavy-interference scenario. As the modules are incrementally incorporated, the metrics SINR, AP, and SSIM exhibit consistent improvements, while MSE and FPR decrease accordingly. This indicates that both the interference robustness and reconstruction quality benefit from the introduction of these components. Specifically, adding the CBAM alone (Model B) already leads to a noticeable performance gain, demonstrating its ability to direct the network’s focus toward interference-sensitive regions. The TE-Net module (Model C) further enhances target contour details, reflected by the improvements in SSIM and AP. Incorporating the IM-Net (Model D) strengthens the model’s generative capability and leads to more realistic reconstructions. The full model (Model E) achieves the best performance across all metrics, confirming the complementary effect of all components.

To further examine the generalization ability under different interference levels, an identical ablation experiment was performed on the mild-interference scenario, and the results show similar performance trends. Table 7 shows the experimental data of specific mild-interference scenarios.

It can be observed that in the mild-interference scenario, the performance improvement trends of each module remain consistent with those in the heavy-interference case, indicating that the modular design of the model provides good adaptability and generalization under different interference conditions. In particular, the introduction of the IM-Net structure significantly enhances the naturalness and consistency of the generated results, while the two-stage architecture ensures that the network can simultaneously handle both interference suppression and target enhancement.

To further assess the computational efficiency and deployment feasibility of the proposed RIME-Net, this section conducts a comprehensive complexity analysis from three perspectives: the number of model parameters (Parameters), floating-point operations (FLOPs), and average inference time (Inference Time). All experiments are performed under the same hardware environment using an NVIDIA RTX 3090 GPU and the PyTorch 2.0 framework. The input RD map size is set to 256 × 256 for fair comparison across all models.

Although RIME-Net consists of two conceptual processing stages, the inference procedure is implemented as a single sequential forward pipeline. TE-Net is intentionally designed to be lightweight, with significantly fewer parameters and computational operations compared to IM-Net. As shown in Table 8, the additional computational overhead introduced by TE-Net is marginal.

Consequently, the total inference time of the complete framework is approximately 16.2 ms per frame, which comfortably satisfies the real-time processing requirements of automotive radar systems, typically constrained to less than 33 ms per frame. Therefore, the proposed two-stage design does not compromise real-time deployability.

From the results in Table 8, it can be observed that although the proposed RIME-Net adopts a two-stage architecture consisting of interference mitigation and target enhancement modules, its overall computational complexity remains only slightly higher than that of representative single-stage networks (e.g., the End-to-End Net). This is mainly attributed to the parameter-sharing strategy and the lightweight architectural design, including the use of 1 × 1 convolutions and the CBAM attention module to enhance channel efficiency.

In addition to computational complexity, RIME-Net exhibits a moderate memory footprint of 67.2 MB, which is comparable to or smaller than that of several benchmark models with similar performance. This indicates that the proposed dual-stage design does not introduce excessive memory overhead and remains compatible with practical embedded deployment constraints.

During inference, the average processing time of RIME-Net is approximately 16.2 ms per frame, which satisfies the real-time requirements of automotive radar perception systems (i.e., less than 33 ms per frame, corresponding to 30 FPS).

Considering computational complexity, memory footprint, and performance metrics jointly, the proposed RIME-Net achieves an effective balance between interference suppression capability and model complexity. This demonstrates that the network is not only suitable for offline signal processing but also has strong potential for deployment on in-vehicle embedded platforms or edge devices.

Therefore, RIME-Net exhibits excellent engineering feasibility in terms of computational efficiency, memory consumption, and real-time performance, providing a valuable reference for the lightweight and real-time design of future millimeter-wave radar perception systems.

## 6. Conclusions

In this study, we proposed RIME-Net, a two-stage unsupervised restoration framework designed for interference suppression and target enhancement in FMCW automotive radar range–Doppler (RD) maps. Unlike previous single-stage or supervision-dependent approaches, the proposed framework integrates a CycleGAN-based IM-Net for global interference mitigation and a lightweight attention-guided TE-Net for local target enhancement. The two-stage architecture effectively decouples low-rank interference removal from sparse target reconstruction, improving both physical interpretability and robustness across diverse operating environments.

Extensive experiments conducted on real-world AWR1642 radar data demonstrate that RIME-Net consistently outperforms classical signal-processing methods and recent deep-learning baselines under light interference, heavy interference, and multi-target scenarios. Quantitative results show significant improvements in SINR, SSIM, AP, and FPR, confirming the superiority of the proposed model in both interference suppression and weak-target preservation. The ablation studies further verify the necessity of each key module—IM-Net substantially improves global structural restoration, TE-Net enhances local target saliency, and their joint optimization yields the best overall performance.

In addition to its accuracy, RIME-Net maintains favorable computational efficiency. The model achieves an average inference latency of ≈16 ms per frame, meeting real-time requirements for in-vehicle radar perception systems. This demonstrates its practical deployment potential on embedded or edge computing platforms.

Overall, the proposed RIME-Net provides a unified, interpretable, and computationally efficient solution for radar interference mitigation and target enhancement. Future work will focus on extending the framework to multi-frame temporal modeling, 3D radar cube processing, and domain-adaptive learning to further improve generalization across varying radar hardware, environments, and traffic conditions.

## Figures and Tables

**Table 1:** Radar hardware parameters.

Parameter	Signal Radar	Interference Radar
Bandwidth (MHz)	2401.44	1619.28
Sweep time (μs)	60	60
Chirp rate (MHz/μs)	40.024	26.988
Sampling rate (ksps)	10,000	10,000

**Table 2:** Simulation parameter settings.

Parameter	Min	Max	Step
Number of targets	1	3	–
Target distance (m)	2	95	–
Interference chirp slope factor	0	1.5	0.1
SNR (dB)	5	40	5
SIR (dB)	−5	40	5

Number of targets and Target distance (m) do not have a Step parameter.

**Table 3:** Quantitative evaluation of interference mitigation under mild interference.

Model	SINR [dB]	MSE	AP [%]	FPR	SSIM
ResNet [1]	20.62	0.0093	86.31	0.0438	0.912
DnCNN [29]	13.68	0.0157	73.81	0.0528	0.874
U-Net [30]	16.31	0.0132	77.28	0.0943	0.891
Autoencoder [19]	16.74	0.0106	81.27	0.0584	0.902
Radar-STDA [24]	17.08	0.0125	82.40	0.0758	0.906
End-to-End Net [14]	19.54	0.0101	85.73	0.0482	0.923
RIME-Net	23.75	0.0084	90.45	0.0375	0.948

**Table 4:** Quantitative evaluation of interference mitigation under severe interference.

Model	SINR [dB]	MSE	AP [%]	FPR	SSIM
ResNet [1]	15.12	0.0138	78.42	0.0674	0.874
DnCNN [29]	10.24	0.0219	66.35	0.0835	0.841
U-Net [30]	12.86	0.0187	70.02	0.1156	0.858
Autoencoder [19]	13.24	0.0159	74.83	0.0893	0.869
Radar-STDA [24]	13.75	0.0174	76.21	0.0962	0.872
End-to-End Net [14]	14.92	0.0148	78.70	0.0715	0.885
RIME-Net	19.48	0.0112	85.16	0.0547	0.918

**Table 5:** Quantitative metrics of different models in multi-target scenarios.

Model	SINR [dB]	MSE	AP [%]	FPR	SSIM
ResNet [1]	18.42	0.0107	83.25	0.0549	0.901
DnCNN [29]	12.32	0.0178	70.56	0.0661	0.868
U-Net [30]	14.95	0.0153	74.03	0.0982	0.882
Autoencoder [19]	15.38	0.0129	78.64	0.0704	0.893
Radar-STDA [24]	15.92	0.0141	80.12	0.0837	0.898
End-to-End Net [14]	17.80	0.0120	83.97	0.0563	0.910
RIME-Net	21.62	0.0098	88.73	0.0451	0.936

**Table 6:** Ablation study results in severe interference scenarios.

Number	Setup	SINR (dB)	MSE	AP (%)	FPR
Model A	Baseline	13.72	0.0184	73.15	0.0813
Model B	+CBAM	15.08	0.0163	76.92	0.0697
Model C	+TE-Net	16.44	0.0151	79.34	0.0625
Model D	+IM-Net	18.57	0.0129	83.11	0.0568
Model E	CBAM + TE-Net + IM-Net	19.48	0.0112	85.16	0.0547

**Table 7:** Ablation study results in mild interference scenarios.

Number	Setup	SINR (dB)	MSE	AP (%)	FPR
Model A	Baseline	16.82	0.0149	80.23	0.0678
Model B	+CBAM	18.07	0.0131	83.51	0.0543
Model C	+TE-Net	19.46	0.0118	86.22	0.0479
Model D	+IM-Net	21.38	0.0096	89.14	0.0421
Model E	CBAM + TE-Net + IM-Net	23.75	0.0084	90.45	0.0375

**Table 8:** Computational cost and runtime comparison of different models.

Model	Parameters (M)	FLOPs(G)	Average Inference Time (ms/Frame)	Memory Footprint (MB)	Explanation
ResNet [1]	11.2	23.4	14.7	44.8	Basic residual structure with few parameters but limited feature expression
DnCNN [29]	8.9	18.6	12.5	35.6	Convolutional stacking is deep, resulting in slower inference
U-Net [30]	18.3	35.1	19.4	73.2	The up- and down-sampling structure is complex, with a large number of parameters
Autoencoder [19]	6.7	12.8	9.6	26.8	Simple structure but limited expressive ability
Radar-STDA [24]	15.5	28.7	17.1	62.0	Rich feature interaction but high computational cost
End-to-End Net [14]	13.9	25.4	15.3	55.6	Balanced performance, moderate inference speed
RIME-Net	16.8	27.9	16.2	67.2	Dual-stage structure but with superior computational efficiency

## Data Availability

The code implementation is publicly available at https://github.com/programmerZhj/radar-denoise-enhance.git (accessed on 3 February 2026).
